# Supplementary material for: Deep learning for bias correction of MJO prediction
Source: Nat Commun. 2021 May 25;12:3087. doi: 10.1038/s41467-021-23406-3 (PMC8149422; doi:10.1038/s41467-021-23406-3)
Supplement: Supplementary file 1 — Supplementary Information [file 41467_2021_23406_MOESM1_ESM.docx]

Supplementary Information for

Deep Learning for bias correction of MJO prediction

H. Kim^1^*, Y. G. Ham^2^, Y. S. Joo^2^, S. W. Son^3^

^1^School of Marine and Atmospheric Sciences, Stony Brook University, New York, US

^2^Department of Oceanography, Chonnam National University, Gwangju, South Korea

^3^School of Earth and Environmental Sciences, Seoul National University, Seoul, South Korea

*Correspondence to: hyemi.kim@stonybrook.edu

Including:

Supplementary Table 1.

Supplementary Fig. 1.

Supplementary Fig. 2.

Supplementary Fig. 3.

Supplementary Fig. 4.

Supplementary Fig. 5.

Supplementary Fig. 6.

Supplementary Fig. 7.

Supplementary Fig. 8.

Supplementary References

**
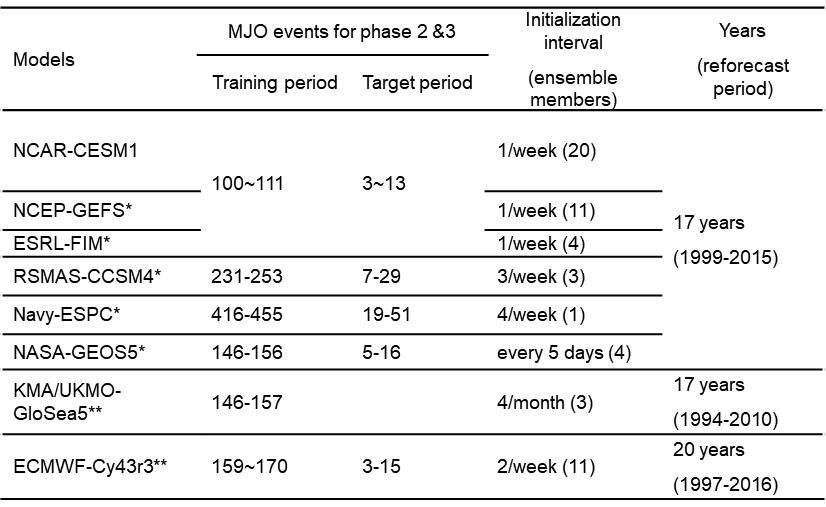
**

**Supplementary Table 1:** **The eight S2S reforecast models considered in this study**. Information includes the number of selected Madden-Julian oscillation (MJO) events for phases 2 and 3, initialization interval, number of ensemble members, years, and reforecast period. One star (*) denotes reforecasts from the Subseasonal Experiment (SubX^1^) projects and from the NCAR Community Earth System Model v1^2^ which follows the SubX protocol, and two stars (**) denote international Subseasonal-to-Seasonal prediction (S2S^3^) models. NCEP-GEFS = National Centers for Environmental Prediction Environmental Modeling Center Global Ensemble Forecast System; NASA-GEOS5 = National Aeronautics and Space Administration Global Modeling and Assimilation Office Goddard Earth Observing System; Navy-ESPC = Naval Research Laboratory Navy Earth System Prediction Capability; RSMAS-CCSM4 = Community Climate System Model version 4 run at the University of Miami Rosenstiel School for Marine and Atmospheric Science; ESRL-FIM = Earth System Research Laboratory Flow-Following Icosahedral Model; NCAR-CESM1 = National Center for Atmospheric Research Community Earth System Model Version 1; KMA/UKMO-GloSea5 = Korea Meteorological Administration-UK Met Office coupled Global Seasonal forecast; ECMWF-Cy43r3: European Centre for Medium-Range Weather Forecasts version Cy43r3.


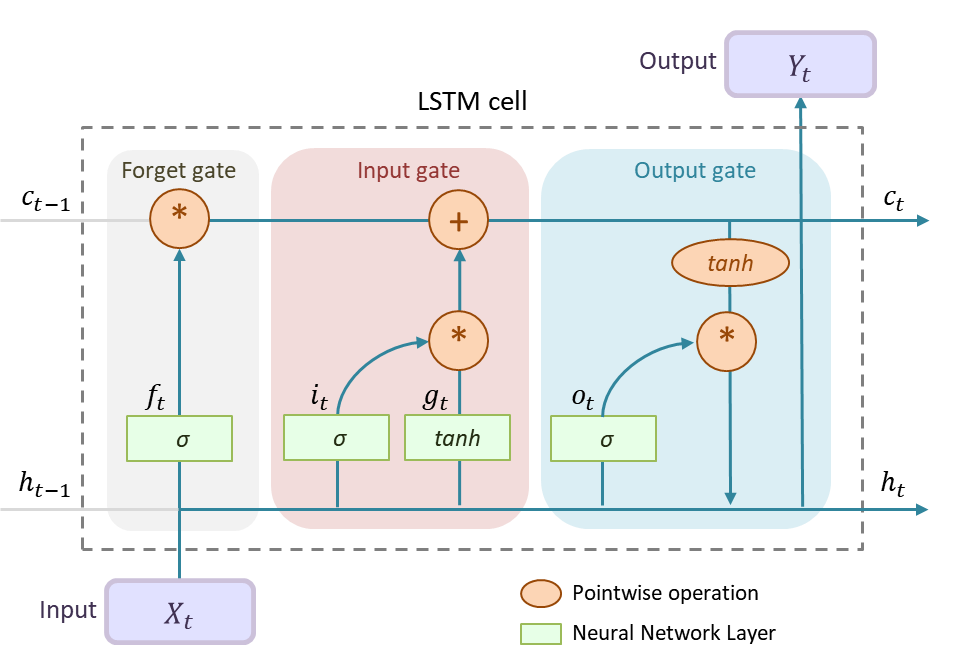


**Supplementary Fig. 1. The architecture of the Long Short-Term Memory (LSTM).** The blue arrows denote the procedures activated in this study, green boxes denote the Neural Network layer, and orange circles denote pointwise operation in forget (*f_t_*), input (*i_t_*), and output (*o_t_*) gates. Input (*X*) and output (*Y*) are marked as violet boxes.


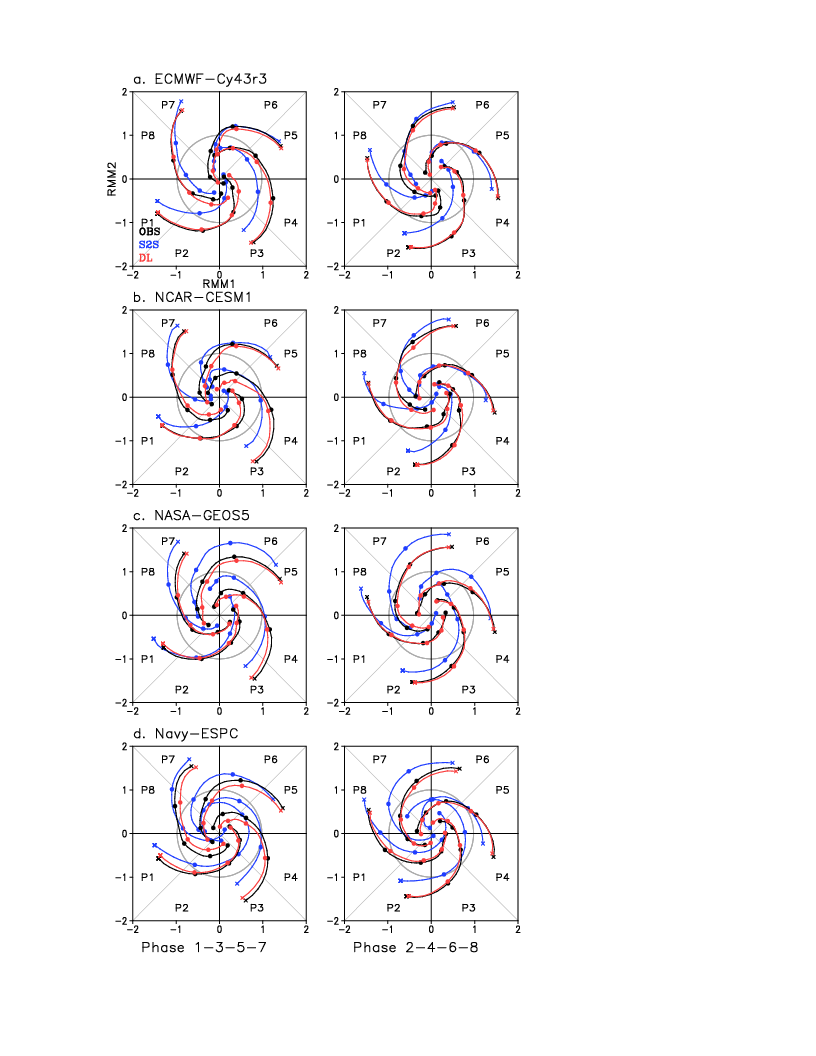

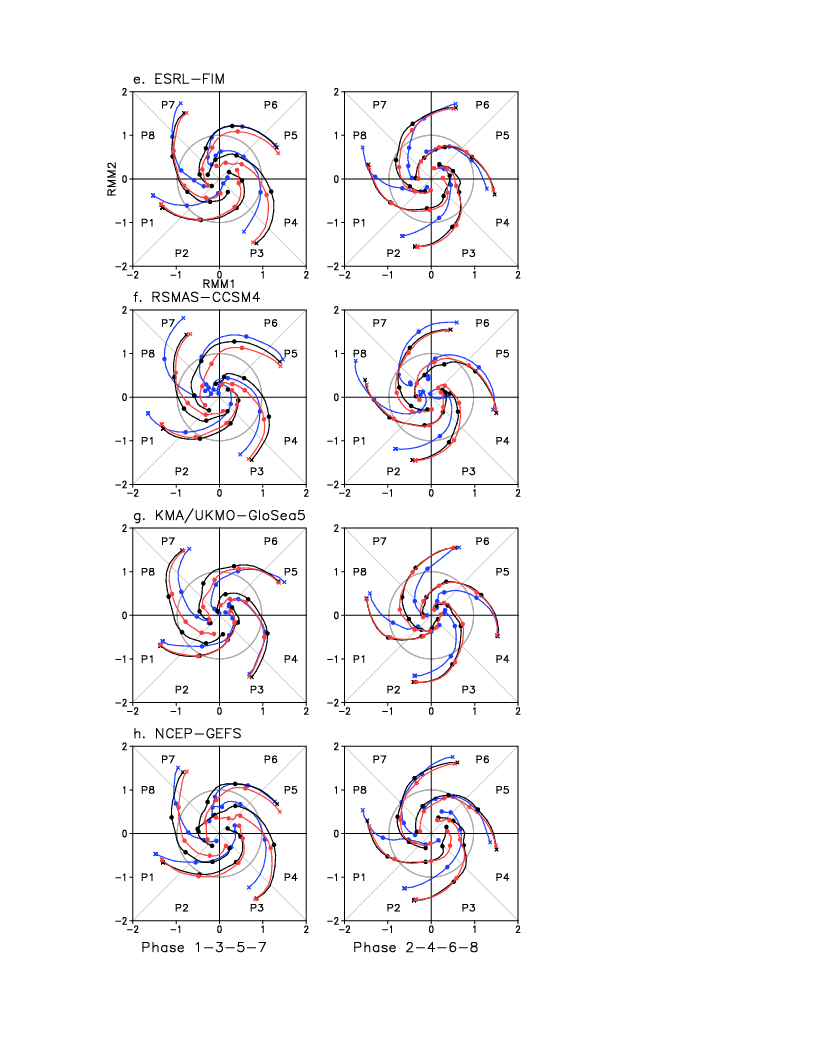


**Supplementary Fig. 2. Observed and predicted Madden-Julian oscillation (MJO) composites for individual models**. Same as Fig. 1, except for (a) ECMWF-Cy43r3, (b) NCAR-CESM1, (c) NASA-GEOS5, (d) Navy-ESPC, (e) ESRL-FIM, (f) RSMAS-CCSM4, (g) KMA/UKMO-GloSea5, (h) NCEP-GEFS. NCEP-GEFS = National Centers for Environmental Prediction Environmental Modeling Center Global Ensemble Forecast System; NASA-GEOS5 = National Aeronautics and Space Administration Global Modeling and Assimilation Office Goddard Earth Observing System; Navy-ESPC = Naval Research Laboratory Navy Earth System Prediction Capability; RSMAS-CCSM4 = Community Climate System Model version 4 run at the University of Miami Rosenstiel School for Marine and Atmospheric Science; ESRL-FIM = Earth System Research Laboratory Flow-Following Icosahedral Model; NCAR-CESM1 = National Center for Atmospheric Research Community Earth System Model Version 1; KMA/UKMO-GloSea5 = Korea Meteorological Administration-UK Met Office coupled Global Seasonal forecast; ECMWF-Cy43r3: European Centre for Medium-Range Weather Forecasts version Cy43r3.


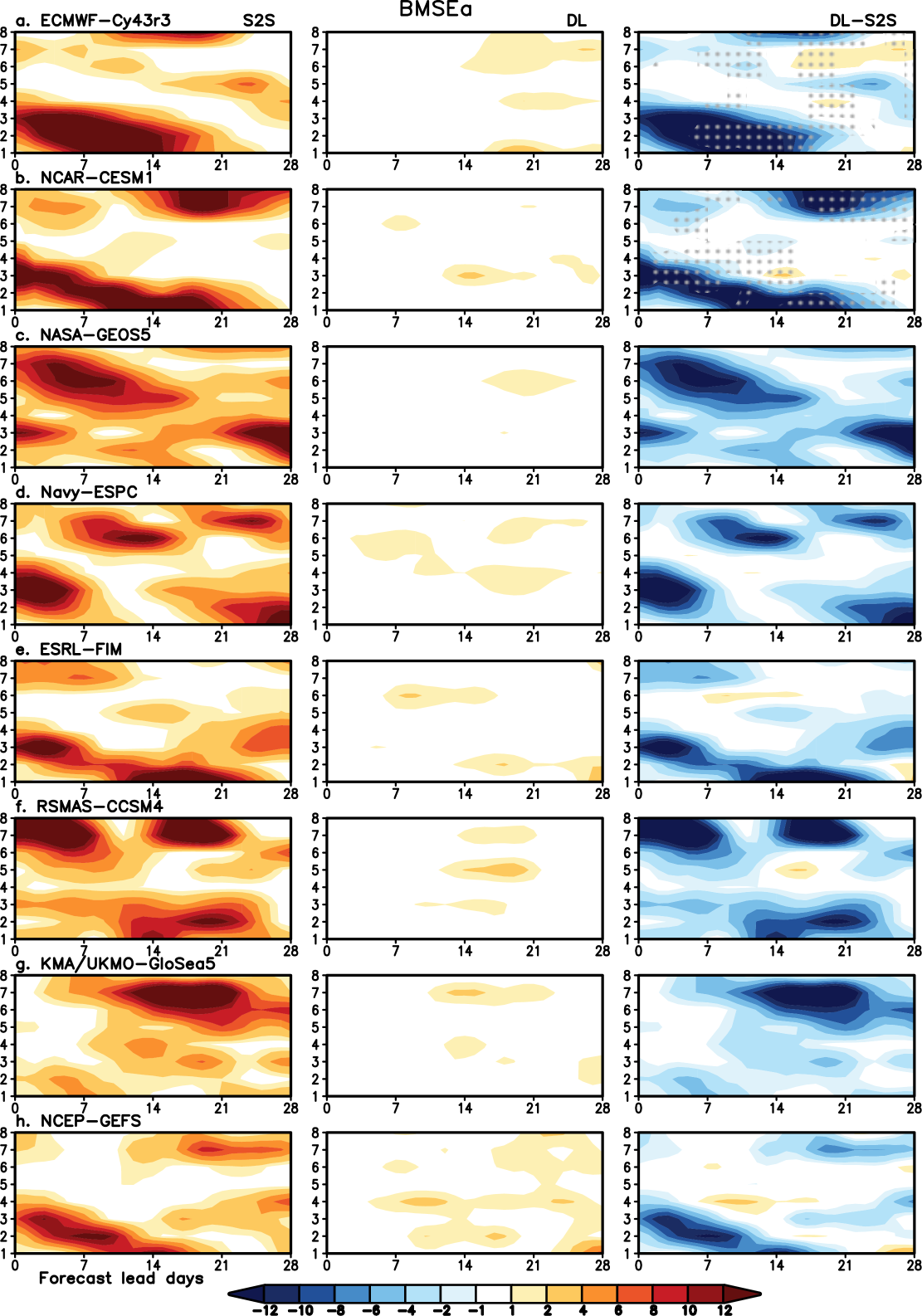


**Supplementary Fig. 3. Bivariate root-mean-squared amplitude error (BMSEa) for individual Subseasonal-to-seasonal (S2S) reforecasts and Deep Learning (DL)-corrections.**  Same as (left) Fig. 2a and (middle) Fig. 2c, except for (a) ECMWF-Cy43r3, (b) NCAR-CESM1, (c) NASA-GEOS5, (d) Navy-ESPC, (e) ESRL-FIM, (f) RSMAS-CCSM4, (g) KMA/UKMO-GloSea5, (h) NCEP-GEFS, and (right) the difference between DL-corrections and S2S reforecasts. In (a) and (b), the stipples mark where BMSEa values from DL-correction are statistically significant at the 95% confidence level. NCEP-GEFS = National Centers for Environmental Prediction Environmental Modeling Center Global Ensemble Forecast System; NASA-GEOS5 = National Aeronautics and Space Administration Global Modeling and Assimilation Office Goddard Earth Observing System; Navy-ESPC = Naval Research Laboratory Navy Earth System Prediction Capability; RSMAS-CCSM4 = Community Climate System Model version 4 run at the University of Miami Rosenstiel School for Marine and Atmospheric Science; ESRL-FIM = Earth System Research Laboratory Flow-Following Icosahedral Model; NCAR-CESM1 = National Center for Atmospheric Research Community Earth System Model Version 1; KMA/UKMO-GloSea5 = Korea Meteorological Administration-UK Met Office coupled Global Seasonal forecast; ECMWF-Cy43r3: European Centre for Medium-Range Weather Forecasts version Cy43r3.


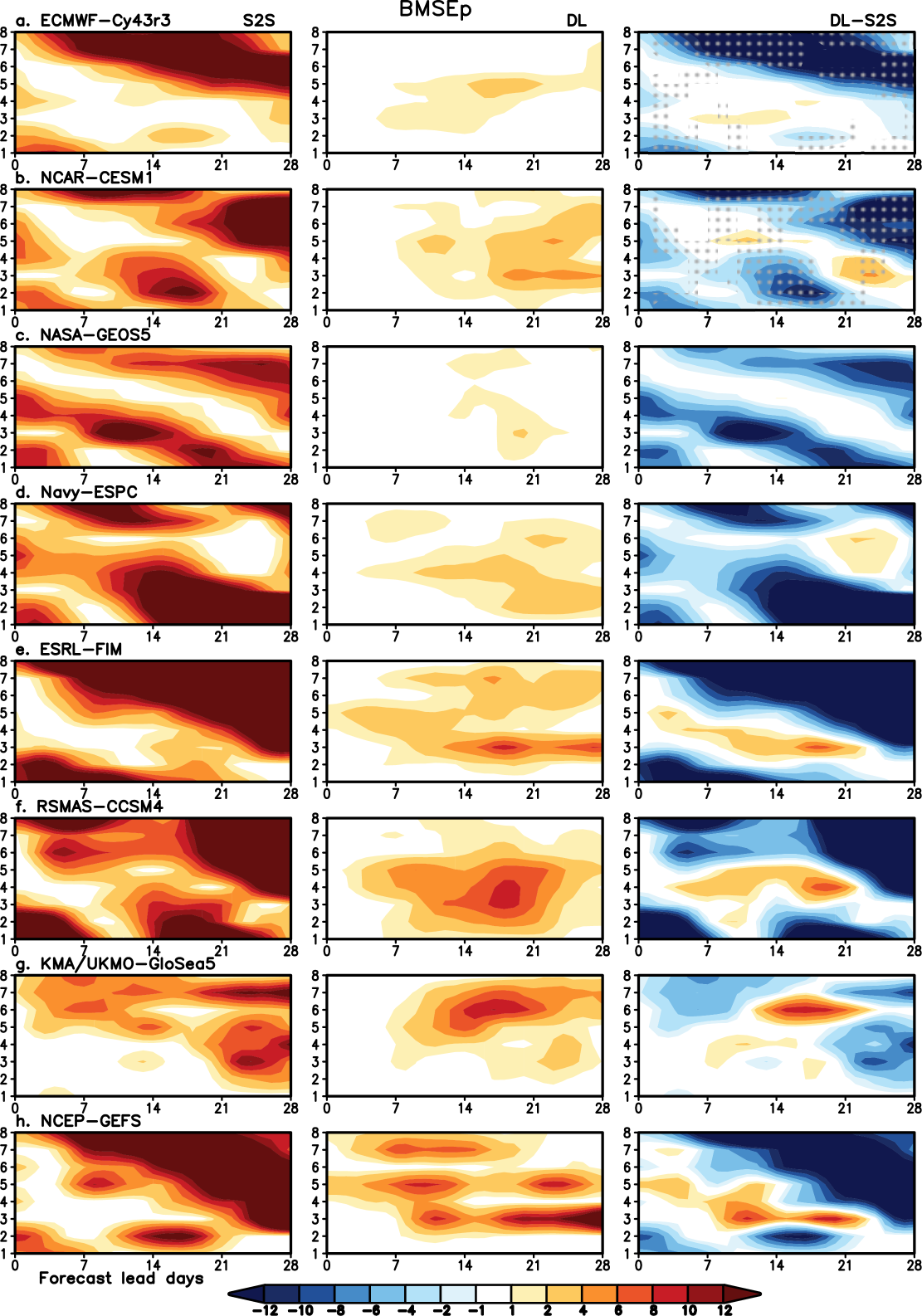


**Supplementary Fig. 4. Bivariate root-mean-squared phase error (BMSEp) for individual Subseasonal-to-seasonal (S2S) reforecasts and Deep Learning (DL)-corrections.**  Same as (left) Fig. 2b and (middle) Fig. 2d, except for (a) ECMWF-Cy43r3, (b) NCAR-CESM1, (c) NASA-GEOS5, (d) Navy-ESPC, (e) ESRL-FIM, (f) RSMAS-CCSM4, (g) KMA/UKMO-GloSea5, (h) NCEP-GEFS, and (right) the difference between DL-corrections and S2S reforecasts. In (a) and (b), the stipples mark where BMSEp values from DL-correction are statistically significant at the 95% confidence level. NCEP-GEFS = National Centers for Environmental Prediction Environmental Modeling Center Global Ensemble Forecast System; NASA-GEOS5 = National Aeronautics and Space Administration Global Modeling and Assimilation Office Goddard Earth Observing System; Navy-ESPC = Naval Research Laboratory Navy Earth System Prediction Capability; RSMAS-CCSM4 = Community Climate System Model version 4 run at the University of Miami Rosenstiel School for Marine and Atmospheric Science; ESRL-FIM = Earth System Research Laboratory Flow-Following Icosahedral Model; NCAR-CESM1 = National Center for Atmospheric Research Community Earth System Model Version 1; KMA/UKMO-GloSea5 = Korea Meteorological Administration-UK Met Office coupled Global Seasonal forecast; ECMWF-Cy43r3: European Centre for Medium-Range Weather Forecasts version Cy43r3.


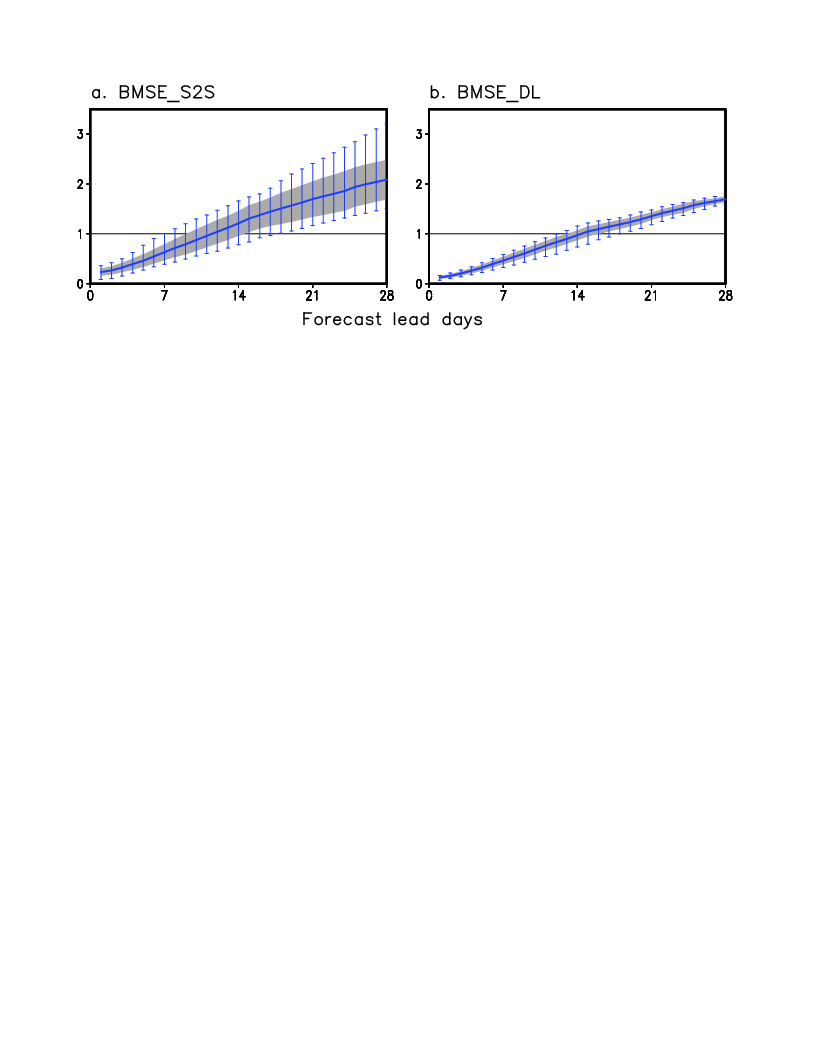


**Supplementary Fig. 5. Averaged forecast errors from individual Madden-Julian oscillation (MJO) events.** Bivariate root-mean-squared error (BMSE) calculated with forecasts of individual MJO events from eight (a) Subseasonal-to-seasonal (S2S) reforecasts and (b) Deep Learning (DL)-corrections averaged over eight phases. The blue contour line represents the average of multi-models, the gray box outlines the ± 1.0 standard deviation of BMSE, and whiskers indicate the minimum and maximum BMSE values among eight different S2S reforecasts. The gray horizontal line is BMSE of 1.0.


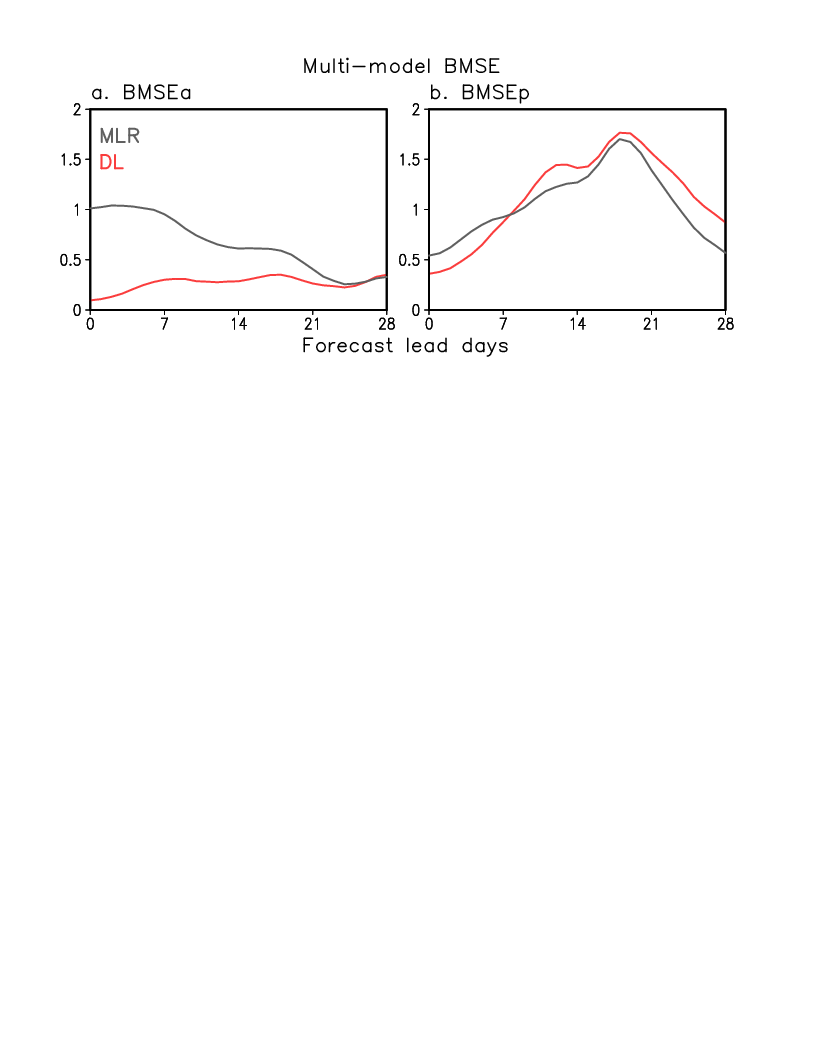


**Supplementary Fig. 6. Forecast errors in Deep Learning (DL)-corrections and Multi-linear Regression (MLR)-corrections.** Same as Fig. 2, except for (a) bivariate root-mean-squared amplitude error (BMSEa) and (b) bivariate root-mean-squared phase error (BMSEp) averaged over eight MJO phases in DL-corrections (red) and MLR-corrections (gray).


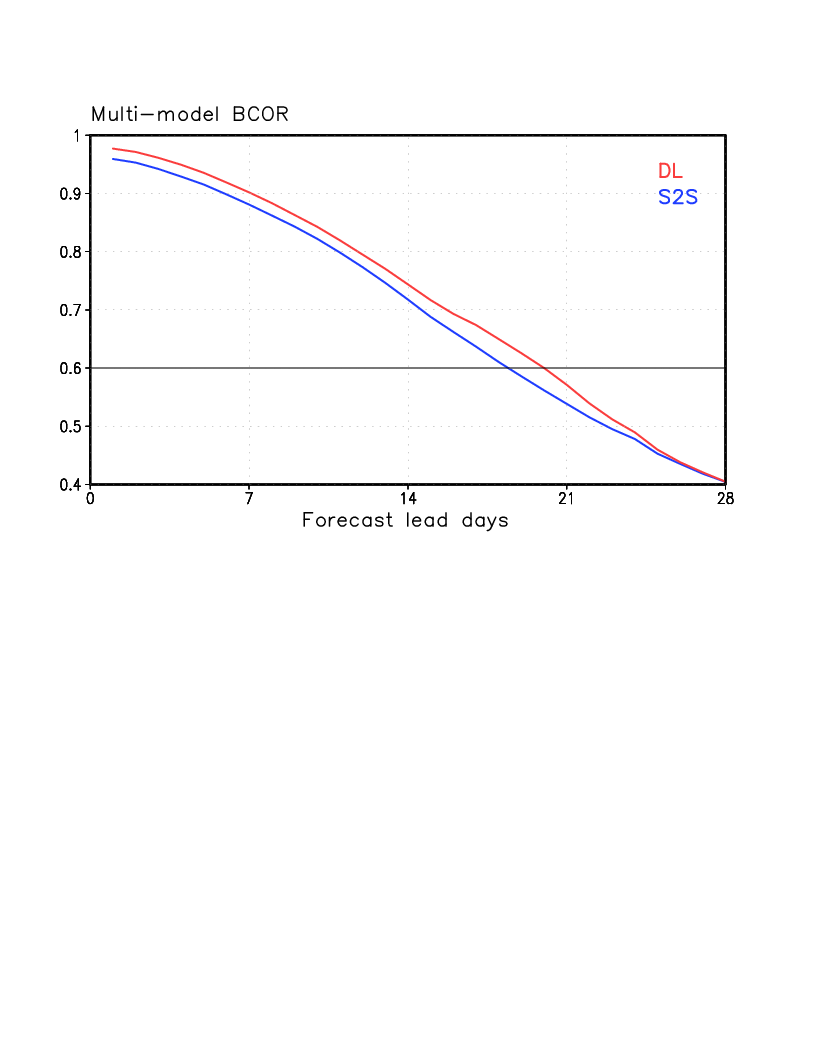


**Supplementary Fig. 7. Correlation coefficients in multi-model mean Subseasonal-to-seasonal (S2S) reforecasts and Deep Learning (DL)-corrections.** Multi-model mean bivariate correlation coefficient (BCOR) of S2S reforecasts (blue) and DL-corrections (red). The gray horizontal line denotes BCOR of 0.6.


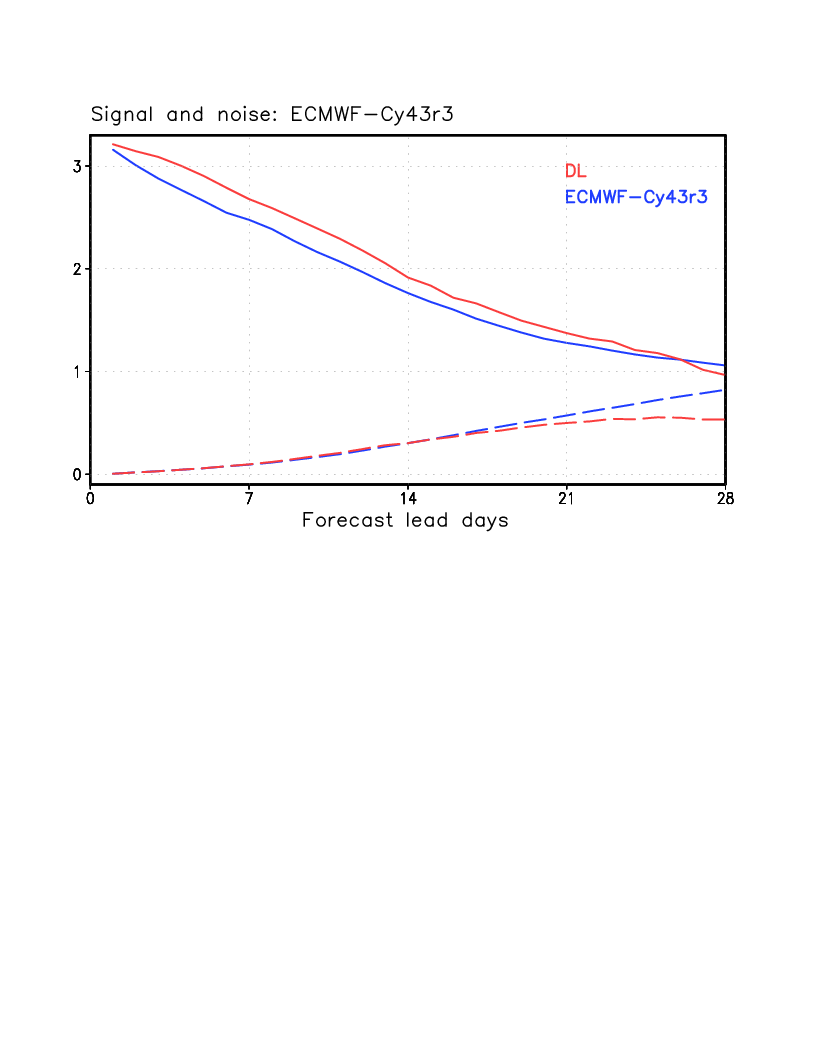


**Supplementary Fig. 8. Forecast signal and noise.** Signal (solid) and noise (dashed) as a function of forecast lead days for the European Centre for Medium-Range Weather Forecasts version Cy43r3 (ECMWF-Cy43r3) (blue) and after Deep Learning (DL)-correction (red).

**Supplementary References**

1. K. Pegion et al., The Subseasonal Experiment (SubX): A Multimodel Subseasonal Prediction Experiment. *B Am Meteorol Soc* 100, 2043-2060 (2019).

2. J. Richter et al, Subseasonal Prediction with and without a Well-Represented Stratosphere in CESM1, *Weather and Forecasting*, 35, 2589-2602 (2020).

3. F. Vitart et al., The Subseasonal to Seasonal (S2S) Prediction Project Database. *B Am Meteorol Soc* 98, 163-173 (2017).
